# Supplementary material for: Metaproteomics as a tool for studying the protein landscape of human-gut bacterial species
Source: PLoS Comput Biol. 2022 Mar 18;18(3):e1009397. doi: 10.1371/journal.pcbi.1009397 (PMC8967034; doi:10.1371/journal.pcbi.1009397)
Supplement: S1 Table — (PDF) [file pcbi.1009397.s002.pdf]

S1 Table: Summary of parent ion charges.

Charge	Count
+1	1
+2	6686036
+3	3528772
+4	566881
+5	40303
+6	5001
+7	15
